# Navigating Challenges in the Management of Mandibular Third Molars With Radix Paramolaris: A Case Report

**DOI:** 10.7759/cureus.45744

**Published:** 2023-09-21

**Authors:** Jay Bhopatkar, Anuja Ikhar, Pradnya Nikhade, Manoj Chandak, Paridhi Agrawal

**Affiliations:** 1 Department of Conservative Dentistry and Endodontics, Sharad Pawar Dental College and Hospital, Datta Meghe Institute of Higher Education and Research (DMIHER), Wardha, IND

**Keywords:** curved canal, extra canal, supernumerary root, radix paramolaris, mandibular third molar

## Abstract

This study aims to shed light on a contemporary approach to preserving third molars instead of opting for immediate extraction. Third molars are known for their diverse shapes and unique anatomy, making root canal treatment a complex task due to limited access. However, there are situations where it is crucial to retain these molars, such as when they provide support or for self-transplantation purposes. The case report focuses on a 33-year-old female patient who presented with pulp necrosis and acute apical periodontitis in the lower right third molar. Instead of extraction, a two-visit conventional root canal treatment was planned. During the initial diagnostic radiographs, only two visible roots were observed, illustrating the typical anatomy of the third molar. However, an unforeseen additional root, referred to as radix paramolaris, was encountered in the mesiobuccal region during the access opening, presenting numerous challenges in the treatment process. Thankfully, advancements in dental technology, such as magnification aids, ultrasonic tips and flexible nickel-titanium (NiTi) rotary files, have rendered the management of such intricate cases more attainable. In conclusion, dealing with intricately curved canals in difficult-to-reach teeth like third molars has become more achievable with technological progress, although the operator's skill and experience remain crucial for effective management.

## Introduction

For a successful root canal treatment, it is necessary to clean and shape the canals efficiently and ensure a good seal with three-dimensional obturation. However, various anatomical and morphological factors can affect the process. Knowing about unusual root canal anatomies is crucial for a successful outcome.

Literature has shown that additional roots are common, with the first reported case of a third root being the radix entomolaris (RE) by Carabelli [1]. These roots are usually found in the distolingual aspect of mandibular first molars, but if they occur in the mesiobuccal area, they are known as radix paramolaris (RP). The characteristic features of these extra roots have been described in detail by Carlsen and Alexanderson [2].

Although third molars also have various anatomical variations, the occurrence of additional roots in these teeth is rare [3]. However, due to the desire of both patients and dentists to retain third molars for future use in autotransplantation or as support for a fixed prosthesis, these teeth are now more often salvaged and root canal treated.

This case report discusses a clinical approach to diagnose and manage a mandibular third molar with three roots, specifically an RP.

## Case presentation

**Figure 1:**
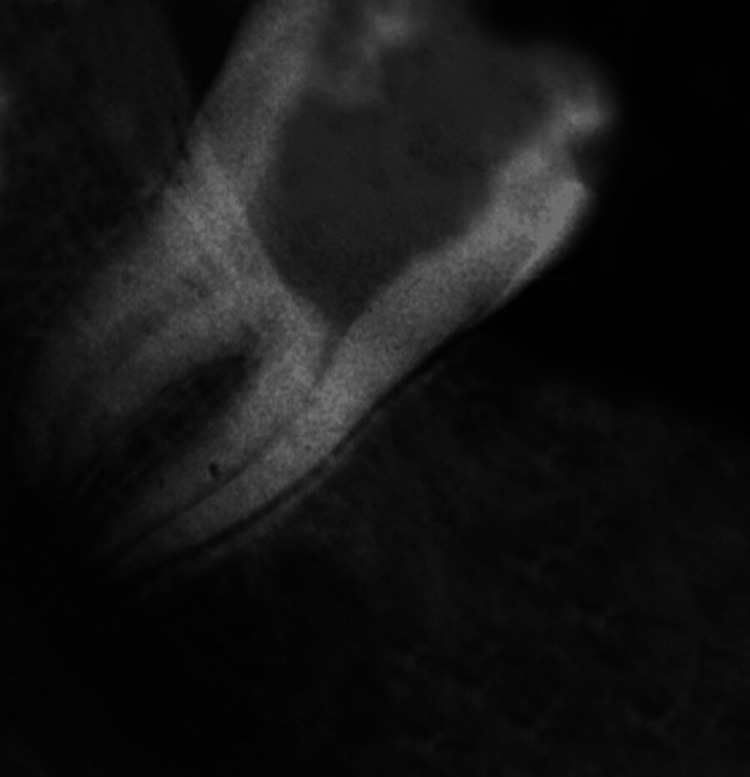
Pre-operative intra-oral radiograph

A 33-year-old female patient reported to the Department of Conservative Dentistry and Endodontics of Sharad Pawar Dental College and Hospital, Wardha, Maharashtra, India with a complaint of pain in the lower-right back region of the jaw for the last 15 days which aggravated in intensity 2-3 days ago. Upon examination, it was found that the lower right third molar had deep occlusal caries and was very tender to percussion. The patient did not have any relevant medical history. The intra-oral periapical radiograph (IOPA) revealed two roots in the lower right third molar one mesial and one distal, but even with angled periapical radiographs, it was impossible to identify the extra root's origin or degree of curvature (Figure 1).

Following the execution of negative responses in both electric pulp testing and cold testing, the diagnosis was made of pulp necrosis with acute apical periodontitis for the lower right third molar. A two-visit root canal treatment was planned.

Treatment

**Figure 2:**
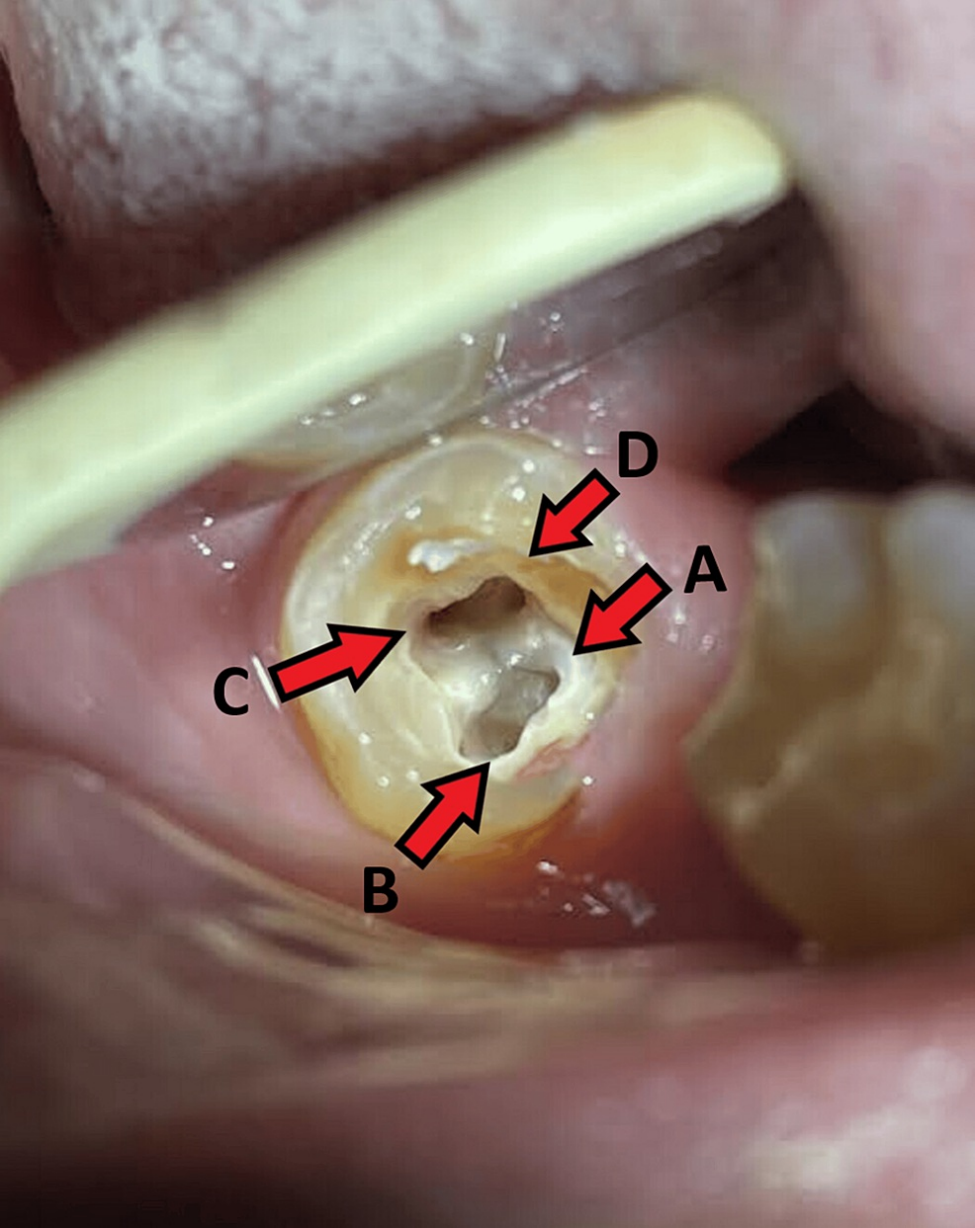
Access opening revealing the distinct presence of all four canal orifices A: Mesio-lingual canal orifice B: Mesio-buccal canal orifice C: Disto-buccal canal orifice D: Disto-lingual canal orifice

Attempting rubber dam placement proved challenging due to several factors, including the mesial tilt of the tooth, restricted mouth opening, the presence of macroglossia, and the absence of the second molar. Consequently, a decision was made to conduct the entire procedure with a single assistant tasked with consistently retracting the tongue using a simple mouth mirror and maintaining suction with a high-powered vacuum suction system, along with the use of cotton rolls. One mesial and two distal canals were located after the initial access cavity was made using a round BR-45 and safe end EX-24 bur (Mani. INC, Japan). Because of the mesial tilt of the tooth and the limited mouth opening of the patient, magnification was attained through the use of magnification loupes (Brilliance loupes, Changzhou Sifary Medical Technology, China), as they appeared to be a more convenient approach than a dental operating microscope. On a closed examination of the pulpal floor, the second grey line running mesiobuccally was seen, raising the possibility of a fourth orifice. The access cavity was subsequently extended more mesiobuccally using ultrasonic tips. Utilizing pre-curved No. 8 and No. 10 C-Pilot files (VDW, Dentsply, USA), root canal exploration and patency checks were conducted. This procedure revealed a new distinct canal orifice in the mesiobuccal region, bringing the total to four (Figure 2). Using the Root ZX Mini apex locator (J.W. Morita, Japan), the working length was established.

After coronal pre-flaring using rotary Hyflex EDM orifice shaper 25/12% (Coltene, Switzerland), all four root canals were prepared using Hyflex CM rotary files up to 25/0.6. Recapitulation with No. 10 K-file (Parcan, Septodont, France) and intermittent irrigation with 5% sodium hypochlorite after each instrumentation was performed. Following the biomechanical preparation, an aqueous calcium hydroxide intracanal medicament (RC Cal, Prime, India) was packed into the canals. This was followed by the insertion of a temporary restorative material (E-Temp, DiaDent, Canada) for seven days.

**Figure 3:**
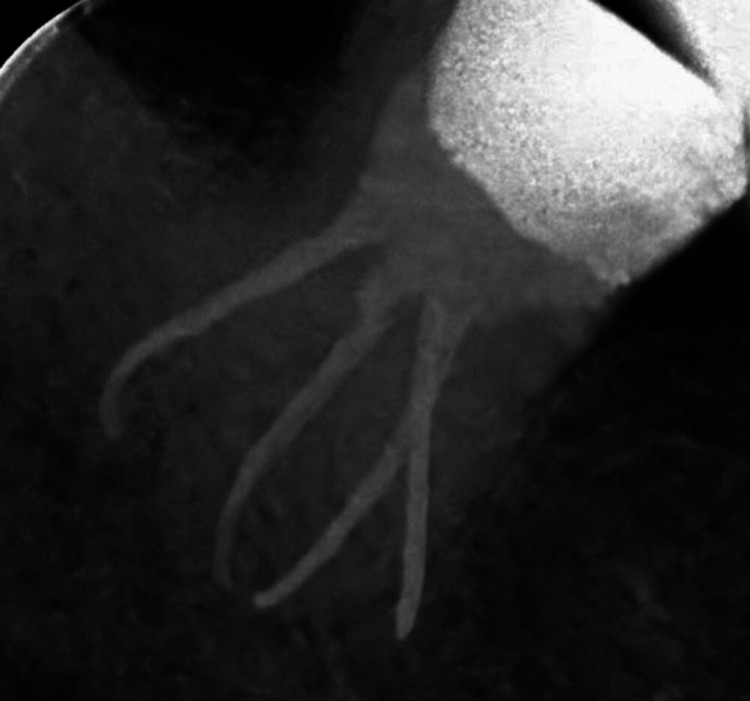
Immediate post-operative radiograph

The molar was asymptomatic at the recall appointment, and the root canals were obturated using matched tapered fitted gutta-percha cones and an epoxy resin-based sealant (Diaproseal, DiaDent, Canada). After that, Flowable glass ionomer cement (iSeal, Prevest DenPro, India) and Filtek Z250 XT (3M, USA) nanohybrid composite were used to restore the access cavity (Figure 3).

## Discussion

It is crucial that clinicians are knowledgeable about the potential for supernumerary roots in mandibular third molars and use appropriate imaging techniques to aid in diagnosis. Two-dimensional imaging alone may not always be sufficient for detecting the presence of supernumerary roots, but the use of periapical radiographs with the tube shift technique or the buccal object rule can be helpful. Clinicians should also be vigilant during clinical inspection of the tooth's crown and cervical morphology for any potential signs of additional roots [4]. The prevalence of supernumerary roots varies depending on the population type, but it is generally low.

In their cone-beam computed tomography (CBCT)-based studies (on populations from Central India and China, respectively), Somasundaram et al. and Wang et al. recorded the highest prevalence of two roots in mandibular third molars. Depending on the population type, the prevalence of three roots may vary from 2% to 5% [5].

The use of C plus files and the HyFlex CM rotary instrument system is a good example of how proper instrument selection can greatly impact the success of root canal treatment, especially in challenging cases with severe canal curvatures. C plus files are stiffer and have an active end-cutting tip, making them ideal for negotiating significantly curved canals. On the other hand, HyFlex CM rotary instruments are extremely flexible and are resistant to cyclic and torsional abuse, which helps to prevent fracture and maintain the original anatomy of the curved canals [6]. The use of these instruments can help to improve the efficiency and safety of root canal treatment in challenging cases.

Yes, there are several challenges associated with performing root canal treatment on third molars. The partial accessibility for instrumentation can make it difficult for the clinician to properly prepare and clean the canals. Additionally, the placement of a rubber dam can be challenging due to the limited space and the position of the tooth. The use of magnification aids can also be limited due to the position of the tooth in the mouth. Furthermore, caution must be exercised when irrigating the canals, as the proximity of the inferior alveolar nerve can increase the risk of periapical extrusion of irrigants. Obtaining radiographs can also be difficult due to the challenging placement of radiographic sensors [7].

The obturation was performed using the single cone technique. While single cone obturation with an epoxy resin-based sealer may not be a novel approach, it can be considered an effective method for canal filling, especially when taking into account the reduced occurrence of voids compared to the cold lateral condensation technique [8].

Absolutely, it is important to assess the level of difficulty and potential risks associated with performing root canal treatment on mandibular third molars before proceeding with the treatment. In addition to the factors mentioned, other considerations such as patient age, medical history, and overall oral health should also be taken into account. Proper case selection, careful planning, and the use of appropriate techniques and materials can help minimize the risk of iatrogenic errors and improve the success rate of the treatment [9]. Close communication with the patient regarding the benefits and risks of the procedure can also help manage their expectations and ensure informed consent.

This case report holds clinical significance by emphasizing the evolving landscape of endodontic care, where preserving third molars, even in cases of intricate anatomies like radix paramolaris, can be a patient-centered and feasible approach, facilitated by technological advancements. It underscores the vital role of dentists' anatomical awareness, radiographic evaluation techniques, and operator expertise in navigating complex root canal treatments while maintaining effective patient communication to ensure informed decision-making.

To summarize the limitations and methods to overcome them, firstly, the limited accessibility and challenging positioning of these molars within the posterior region of the oral cavity can pose difficulties in both diagnosis and treatment planning. Overcoming this limitation can involve the utilization of advanced imaging techniques, such as CBCT, to provide a comprehensive three-dimensional view of the tooth's anatomy and its proximity to vital structures like the inferior alveolar nerve. Additionally, the potential presence of complex root canal anatomies, such as radix paramolaris, as highlighted in this case report, necessitates careful consideration during treatment. The use of flexible nickel-titanium rotary files, magnification aids, and ultrasonic tips can enhance operator precision when navigating intricate root canal systems. Moreover, issues related to patient cooperation, including limited mouth opening and discomfort, can hinder successful treatment. To address these concerns, practitioners can employ appropriate behavior management techniques, including the use of an effective anesthesia protocol, patient communication, and the provision of a comfortable environment to ensure patient comfort and cooperation. By acknowledging and proactively addressing these limitations through advanced diagnostic tools, specialized instrumentation, and patient-centered strategies, dental practitioners can optimize the management of mandibular third molars, thereby improving treatment outcomes and patient satisfaction.

## Conclusions

In conclusion, this case report illuminates the clinical complexities and treatment considerations associated with preserving third molars, specifically in the presence of a radix paramolaris. It underscores the importance of anatomical proficiency, radiographic precision, and operator expertise in successfully managing challenging root canal treatments. Moreover, it highlights the evolving paradigm in endodontics that places patient-centered care at the forefront, enabling the preservation of third molars for various clinical purposes when aligned with patient goals. Technological advancements have played a pivotal role in enhancing the feasibility of such treatments. Nonetheless, it is imperative for dental practitioners to exercise prudence, skill, and comprehensive patient communication to make informed decisions and achieve favorable outcomes in these intricate cases. This report contributes to the expanding body of knowledge in endodontics and serves as a testament to the continuous evolution of dental practices to align with the unique needs and preferences of patients.
